# Should the management of high grade cervical squamous intraepithelial lesion (HSIL) be different in HIV-positive women?

**DOI:** 10.1186/s12981-021-00371-x

**Published:** 2021-07-23

**Authors:** Christine Gilles, Maude Velghe-lenelle, Yannick Manigart, Déborah Konopnicki, Serge Rozenberg

**Affiliations:** 1 Department of Obstetrics and Gynecology, Saint Pierre University Hospital Brussels, Université Libre de Bruxelles (ULB), Free University of Brussels (ULB-VUB), 322 Rue Haute, 1000 Brussels, Belgium; 2 Infectious Disease Department, Saint-Pierre University Hospital Brussels, Université Libre de Bruxelles (ULB), Brussels, Belgium

**Keywords:** HIV, HSIL, Treatment failure, CIN

## Abstract

**Background:**

This study compares the management and outcome of high grade squamous intraepithelial lesions (HSIL) in HIV-positive and -negative women and identifies risk factors for treatment failure.

**Methods:**

This retrospective, controlled study includes 146 HIV-positive women, matched for HSIL, age and year of diagnosis, with 146 HIV-negative women. Differences were analysed using parametric and non-parametric tests and Kaplan–Meier survival curves. A binary logistic regression was used to assess risk factors for treatment failure.

**Results:**

Persistence of cervical disease was observed most frequently in HIV-positive women (42 versus 17%) (p  <  0.001) and the cone biopsy margins were more often invaded in HIV-positive-women than in HIV-negative ones. (37 versus 16%; p  <  0.05).

HIV-positive women, with successful cervical treatment had better HIV disease control: with significantly longer periods of undetectable HIV viral loads (VL) (19 versus 5 months; p  <  0.001) and higher CD4 counts (491 versus 320 cells/mm^3^; p  <  0.001). HIV-positive women with detectable VL at the time of dysplasia had 3.5 times (95% IC: 1.5–8.3) increased risk of treatment failure. Being treated through ablative therapy was associated with a 7.4, four-fold (95% IC: 3.2–17.3) increased risk of treatment failure compared to conization

**Conclusion:**

HIV-positive women have a higher risk of treatment failure of HSIL than do HIV-negative women, especially when ablative therapy is used and in women with poor control of their HIV infection. The management and the follow- up of HSIL’s guidelines in this high-risk population should be adapted consequently: for HIV-positive women with uncontrolled viral load, excisional treatment should be the preferred therapy, whereas for women with undetectable viral load, CD4  +  lymphocytes higher than 500 cells/mm^3^ and with a desire of pregnancy, ablative therapy may be considered.

## Background

It was estimated in 2018, that 40 million people worldwide were living with HIV, of which about half were women [1]. The same year, it was estimated that there were 570,000 new cases of cervical cancer, which represents 6.6% of all female cancers, making it the fourth most frequent cancer affecting women [2].

In 1993, the Centre for Disease Control and Prevention (CDC) concluded that HIV infected women, diagnosed with moderate or severe cervical intraepithelial neoplasia (CIN), should be viewed as suffering from an early stage, symptomatic HIV infection (category B), and those with invasive cervical cancer should be viewed as suffering from acquired immunodeficiency (AIDS; category C) [3]. Being infected with HIV is a risk factor for acquiring HPV infection, developing a persistence of HPV with subsequent CIN, or even for cervical cancer [4, 5]. Consequently, HIV-positive women have on average a three times higher incidence of cervical lesions than HIV-negative women [6].

In 2005, we compared HIV-positive and negative women after treatment of CIN 2 and CIN 3 [CIN2  +  or high grade intraepithelial squamous lesions (HSIL)] and reported that HIV positive women had higher rates of recurrence of CIN after a median follow-up of 22 months [7], which was confirmed in a systematic review [8].

At that time, many patients were not optimally treated as they were not using Combined Antiretroviral Therapy (cART), due to compliance issues (such as multiple pills and intakes or side effects). Moreover, many patients had been treated for only short periods of time [7]. Since then, compliance friendly cART (including one pill per day) has become widely available [9].

The primary aim of this study is to compare the management and the outcome of HSIL, for HIV-positive and HIV-negative women. The secondary aims were to address failure and success differences in relation to the chosen treatment (conization which refers to the excision of a cone-shaped portion of the cervix versus ablative therapy which refers to cryotherapy or electrocoagulation) and to determine risk factors for failures within the group of HIV positive women.

## Materials and methods

### Design

We conducted a retrospective matched controlled study.

### Selection of the population

**Fig. 1:**
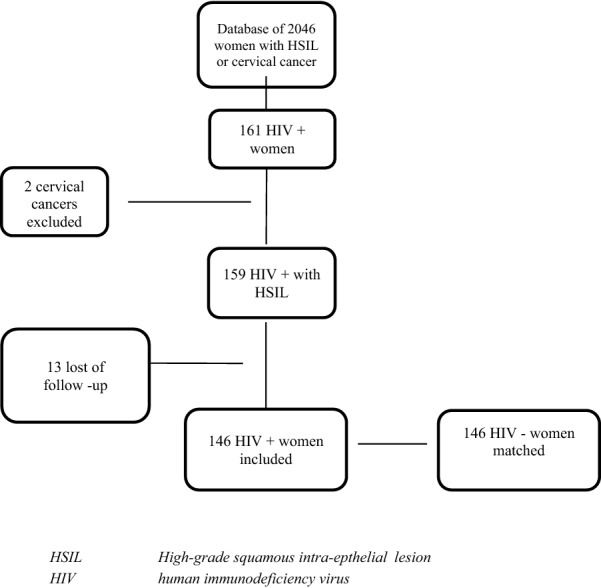
Flow chart of the patient’s selection: using the pathology data of all the patients diagnosed, with CIN 2 or CIN 3 (CIN2  + /high grade squamous intraepithelial lesion), between January 2003 and December 2017 (n  =  2669), we included finally 146 HIV  +  women. We matched (matching criteria: CIN2  + , age, and year of diagnosis) 146 HIV-women from the pathology databank. All the women needed to have at least one follow-up cervical smear after management

Using the pathology data bank from our hospital, we identified all patients who had been diagnosed with CIN2  +  (CIN 2 or CIN 3 or both, defined as HSIL) at biopsy or from conization specimens, between January 2003 and December 2017 (n  =  2669). We cross-matched this list with the data-bank of our HIV reference centre, identifying HIV positive women (n  =  161). Fifteen patients were excluded (3 who had cervical cancer and 12 with no follow-up, resulting in the selection of 146 HIV positive women who were included in this study (Fig. 1). We matched them with HIV negative women, found in the initial data-base, using the following matching criteria: CIN2  + , age, and year of diagnosis and without knowledge of any other item. At least one follow-up cervical smear after management was required for all the patients (Fig. 1).

### Data collection

We collected the following socio-demographical data from the computerised medical files: ethnic origin, access to health coverage, tobacco use, obstetrical history and parity, past and present contraceptive use and method “[IUD, combined hormonal contraception (pill, patch, ring), progestogen containing contraception (pill, injectable, implant), condom]”.

STI history (syphilis, herpes, chlamydia trachomatis, neisseria gonorrhoea, hepatitis b), HPV vaccination history.

### Data pertaining to the cervical lesion

Date of treatment, type of treatment (follow-up, cone biopsy or also known as conization (all excisional procedures were performed using loop electrosurgical excision procedure (LEEP), local treatment (application of trichloroacetic acid, imiquimod or podophylin), ablative therapy (cryotherapy, electrocoagulation) hysterectomy), the pathology results of the specimen (normal, low grade squamous intraepithelial lesion, LSIL, or HSIL for high grade squamous intraepithelial lesion), characteristics of conization specimen (whether the margins were free of disease or not (without distinction of whether it was endocervical or exocervical), its size, weight and volume), HPV genotyping results when available, it was expressed differently over time: either expressed as HRHPV  +  (if any of the following was positive 16/18/31/33/35/39/45/51/52/56/58/59/68) without precise identification of the genotype by Hybrid captur, (hc2 High-Risk HPV DNA Test, Digene^®^, USA) or either expressed as HPV 16 or 18  +  or other than 16 and 18 (31/33/35/39/45/51/52/56/58/59/68) performed by PCR (Abbott HRHPV^®^).

### Follow-up data

Date and result of every follow-up cervical smear, HPV testing and/or cervical biopsy results, duration of the follow-up (in weeks). We defined persistence as the presence of an abnormal smear (ASCUS HPV HR  + , LSIL, HSIL) at the first follow- up visit. We defined a recurrence as being an HSIL lesion diagnosed by biopsy, during follow-up after having had a previous, normal cervical result. We defined as treatment failure when a persistence or a recurrence was observed.

### HIV infection data

Age at time of HIV diagnosis, CD4 count rate (cells/mm^3^) and viral load (copies/ml) at time of HIV diagnosis and at time of cervical lesion diagnosis, risk factors for HIV transmission (heterosexual, intravenous drug use, transfusion, mother-to-child), cART type (Nucleoside analog reverse-transcriptase inhibitors, Protease Inhibitors, Non-nucleoside reverse-transcriptase inhibitors, Integrase Inhibitors)and intake., duration of cART until the dysplasia was discovered (length of time during which cART was used in months) and effective duration of cART taking into account treatment compliance (cumulative length of time during which cART was used in months), AIDS stage according to the CDC classification (A, B, C), CD4/CD8 at the time of cervical lesion diagnosis; maximum viral load (copies/ml) and CD4 nadir (cells/mm^3^) between HIV diagnosis and cervical lesion diagnosis; cumulative time of viral load  <  50 copies/ml.

### Statistical analyses

Descriptive statistics were analysed. Normal distribution was checked by visual histograms and using the Shapiro–Wilk test (p  <  0.05). Differences in the distribution of variables were tested for independent groups using the Student T test that were normally distributed or using Wilcoxon–Mann–Whitney test for variables that were not normally distributed, since we were unable to match the patients for ethnicity. Differences in proportions were tested using Khi Square teste and exact de Fisher correction for small numbers; Kaplan–Meier analysis was used to assess persistence and recurrence in relation to time (days without recurrence or persistence). Differences between groups were tested using the Log-rank test.

A binary logistic regression was used to assess risk factors of persistence, expressed as Odd Ratios (OR) with a 95% confidence interval. A generalized linear regression model was used to assess the analysis of variance for multiple dependent variables. Cox-regression was used to estimate Hazard ratios.

All analyses were performed using SPSS (IBM statistics 25) or SAS.

The study was approved on March 20th, 2018 by the Saint-Pierre University Hospital Ethical Review Board (nr CE/18-03-08).

## Results

### ***Population characteristics ***(Table 1)

**Table 1:** Demographic and clinical data of HIV negative and positive women with HSIL

	Evaluated population (total N = 292)^c^	HIV − (N = 146)	HIV + (N = 146)	P value
Age				
Median (IQR)	292	35 (29–41)	35 (29–41)	0.944^a^
Origin	269	N = 123	N = 146	< 0.001^b^
Europe	92	76 (61.8%)	16 (10.9%)	
North Africa	30	29 (23.6%)	1 (0.7%)	
Sub-Sahara Africa	140	12 (9.8%)	128 (87.7%)	
Asia	1	1 (0.8%)	0 (0%)	
South America	6	5 (4.1%)	1 (0.7%)	
Level of education	76	N = 18	N = 58	0.058^b^
Primary school	15	3 (16.7%)	12 (20.7%)	
Secondary school	36	5 (27.8%)	32 (53.4%)	
Higher education	25	10 (55.6%)	15 (25.7%)	
Employment	141	N = 42	N = 99	< 0.001^b^
Unemployed	85	14 (33.3%)	71 (71.7%)	
Employed	49	25 (59.5%)	24 (24.2%)	
Student	4	2 (4.8%)	2 (2%)	
Retired	3	1 (2.4%)	2 (2%)	
Legal status	227	N = 83	N = 144	0.011^b^
Legally in Belgium	196	78 (94%)	118 (81.9%)	
No residence permit	31	5 (6%)	26 (18.1%)	
Access to health care	288	N = 142	N = 146	< 0.001^b^
None	11	1 (0.7%)	10 (6.8%)	
Medical regular insurance	237	130 (91.5%)	107 (73.3%)	
Social assistance	40	11 (7.7%)	29 (19.9%)	
Smoking	258	N = 121	N = 137	< 0.001^b^
Non-smoker	176	65 (53.7%)	111 (81%)	
Smoker	82	56 (46.3%)	26 (19%)	
Contraception	270	N = 138	N = 132	< 0.001^b^
None	122	62 (44.9%)	60 (45.5%)	
Hormonal contraception	80	51 (36.9%)	29(21.9%)	
Non hormonal contraception	68	25 (18.2)	43 (32.6)	
Past history of STI	290	N = 144	N = 146	< 0.001^b^
Not reported	244	138 (95.8%)	106 (72.6%)	
At least one episode of STI	46	6 (4.2%)	40 (27.4%)	
Parity	289	143	146	0.024^a^
Median (IQR)		1 (0–2)	1 (1–3)	

Hormonal contraception includes estro-progestins (pill, ring, patch), progestin methods include (progestin only pill, implant, injectable) and the use of concomitant hormonal method and condoms

Non hormonal contraception includes IUD, condoms and sterilization

*HIV* human immunodeficiency virus; *STI* sexually transmitted infection; *HSIL* high grade squamous intraepithelial lesion; *IQR* interquartile range

^a^Mann–Whitney test

^b^khi-square test

^c^Some analyses were realised on a restricted number of patients. These numbers are provided in the column evaluated population

The HIV infected women who came mostly from sub-Saharan Africa, were more frequently unemployed, had less health-insurance coverage, were less educated and smoked less than HIV-negative women (Table 1). Twenty-six percent of HIV-positive women used condoms compared to 3% of the negative ones. There was a higher prevalence of sexually transmitted infections in the group of HIV positive women (27%) than in HIV negative ones (4%).

### ***Cervical lesion data ***(Table 2)

**Table 2:** Cervical lesion, management and follow up of HIV negative and positive women

	Evaluated population N = 292^d^	HIV − (N = 146)	HIV + (N = 146)	P value
Baseline pap-test	286	N = 140 (100%)	N = 146 (100%)	0.010^b^
Normal	1	0 (0%)	1 (0.7%)	
LSIL	90	38 (27.1%)	52 (35.6%)	
HSIL	152	73 (52.1%)	79 (54.1%)	
ASCUS	35	22 (15%)	14 (9.6%)	
ASCH	7	7 (5%)	0 (0%)	
Cancer	1	1 (0.7%)	0 (0%)	
HPV	141	N = 42	N = 99	0.325^b^
Negative	9	2 (4.8%)	7 (7.1%)	
HR HPV	108	31 (73.8%)	77 (77.8%)	
HPV 16/18	8	5 (11.9%)	3 (3%)	
HPV other than 16/18	13	3 (7.1%)	10 (10.1%)	
HPV 16 and other	3	1 (2.4%)	2 (2%)	
Baseline biopsy	284	N = 140 (100%)	N = 144 (100%)	0.856^b^
Normal^c^	4	2 (1.4%)	2 (1.4%)	
LSIL^c^	3	2 (1.4%)	1 (0.7%)	
HSIL	277	136 (97.1%)	141 (97.9%)	
Vaccination	280	N = 134	N = 146	0.791^b^
Unvaccinated	80	40 (29.8%)	40 (27.4%)	
Vaccinated	17	9 (6.7%)	8 (5.5%)	
Not reported	183	85 (63.4%)	98 (67.1%)	
Treatment	292	N = 146 (100%)	N = 146 (100%)	0.290^b^
Follow-up	27	12 (8.2%)	15 (10.3%)	
Conisation	172	94 (64.4%)	78 (53.4%)	
Ablative treatment or local therapy	85	37 (25.3%)	48 (32.9%)	
Hysterectomy	8	3 (2%)	5 (3.5%)	
Persistence	292	N = 146 (100%)	N = 146 (100%)	< 0.001^b^
No	207	122 (83.5%)	85 (58.2%)	
Yes	85	24 (16.5%)	61 (41.8%)	
Type of persistence	85	N = 24 (100%)	N = 61 (100%)	0.072^b^
CIN1	32	9 (37.5%)	28 (45.9%)	
CIN2/CIN3	35	10 (41.6%)	29 (47.5%)	
ASCUS HPV +	5	4 (16.6%)	1 (1.6%)	
ASCH	4	1 (4.3%)	3 (5%)	
Recurrence	292	N = 146 (100%)	N = 146 (100%)	0.18^b^
No	277	136 (93.1%)	141 (96.5%)	
Yes	15	10 (6.9%)	5 (3.5%)	
Time of follow-up (in weeks)				
Median (IQR)	292	142 (62–304)	165 (48–444)	0.112^a^

*HIV* human immunodeficiency virus; *HPV* human papillomavirus; *LSIL* low grade squamous intraepithelial lesion; *HSIL* high grade squamous intraepithelial lesion; *ASCUS* atypical squamous cell of undetermined significance; *ASCH* atypical squamous cells that cannot exclude HSIL; *HRHPV* high risk human papillomavirus; *IQR* interquartile range; *CIN* cervical intraepithelial neoplasia

^a^Mann–Whitney test

^b^khi-carré test

^c^These patients had HSIL diagnosis at time of conization

^d^Some analyses were realised on a restricted number of patients. These numbers are provided in the column evaluated population

There were no differences between the two groups in terms of HSIL management: respectively 53% of HIV-positive and 64% of HIV-negative women underwent a cone biopsy and 33% of HIV positive and 25% of HIV negative women were treated by topical therapy (which includes ablative therapy or local application of trichloroacetic acid, podophylin or imiquimod). Persistence of cervical disease was more frequently observed in HIV-positive women (41.8%, of which 47.5% had a HSIL lesion) than in HIV negative ones (16.5%, of which 41.6% had a HSIL; p  <  0.001). Median time to persistence was 63 days (IQR: 6–172). There was no difference between the two groups in terms of recurrence.

The duration of follow- up was comparable in the two groups (165 weeks in HIV-positive versus 142 weeks in HIV-negative). The number of follow-up visits were the same between the two groups with a median of 7 (IQR: 1–17). Seventeen women (9 HIV-negative, 8 HIV-positive) were vaccinated against HPV (details were missing about the vaccine type); this data was missing for 183 patients.

### ***Cone biopsy data ***(Table 3)

**Table 3:** Data concerning the conisation specimen in HIV  +  and  −  women

	N = 172	HIV − (N = 94)	HIV + (N = 78)	P value
Pathology report	170	N = 92 (100%)	N = 78 (100%)	0.353^b^
Free of disease	7	2 (2.2%)	5 (6.4%)	
LSIL	8	5 (5.4%)	3 (3.8%)	
HSIL	155	85 (92.4%)	70 (89.7%)	
Excision margins	171	N = 93 (100%)	N = 78 (100%)	0.002^b^
Free of disease	127	78 (83.9%)	49 (62.8%)	
Involved	44	15 (16.1%)	29 (37.2%)	
Weight of the specimen (gr)	56	N = 35	N = 21	
Median (IQR)	2.48 (1.8–3.9)	2.4 (1.77–4.5)	2.55 (1.91–3.36)	0.774^a^
Depth of the specimen (cm)	166	N = 92	N = 74	
Median (IQR)	1.2 (1–1.6)	1.25 (1–1.6)	1.15 (1–1.52)	0.58^a^
Volume the specimen (cm^3^)	166	N = 92	N = 74	0.58^a^
Median (IQR)	4.2 (2.45–6.81)	4.5 (2.47–7.34)	4 (2.4–5.8)	

*LSIL* low grade squamous intraepithelial lesion; *HSIL* high grade squamous intraepithelial lesion

^a^Mann–Whitney test

^b^khi-Square test

High grade lesions were confirmed in cones, respectively, in 90 and 92% of the specimens from HIV  +  and HIV  −  women. The margins of the specimens were more frequently positive in HIV-positive women than in HIV negative ones (37 versus 16%; p  <  0.05). There were no differences in volume, depth nor weight of the biopsy specimens between the two groups.

### ***Characteristics of the HIV-positive women ***(Table 4)

**Table 4:** Characteristics of the HIV infection at time of cervical dysplasia diagnosis in relationship with cervical treatment failure

	HIV (all women) N = 146	HIV women with successful SIL treatment N = 80	HIV women with SIL treatment failure N = 66	P value
Age at HIV diagnosis				
N	146	80	66	
Mean (SD)	28	29.5 (7.7)	27.8 (8.1)	0.47^a^
Risk factor for HIV acquisition				
N	137	75 (100%)	62 (100%)	0.09^b^
Blood transfusion	2	0	2 (3.2%)	
Heterosexual	130	74 (98.7)	56 (90.3)	
IVD	2	1 (1.3%)	1 (1.6%)	
Mother to child	3	0	3 (4.8%)	
cART use	146	80 (100%)	66 (100%)	
Yes	124 (84.9%)	69 (86.3%)	55(83.3%)	0.62^b^
No	22 (15.1%)	11(13.7%)	11 (16.7%)	
Duration of time using cART at time of dysplasia (month)				
N	124	69	55	
Median (IQR)	34 (10–85)	35 (11–82)	27 (8–104)	0.95^a^
Cumulative duration of time treated with cART (month)				
N	124	69	55	
Median(IQR)	23 (7–60)	29 (9–62)	22 (6–61)	0.53^a^
HIV classification (CDC)	146	80 (100%)	66 (100%)	
A and B	124 (84.9%)	70 (87.5%)	54 (81.8%)	0.34^b^
C	22 (15.1%)	10 (12.5%)	12 (18.2%)	
VL at time of dysplasia diagnosis (copies/ml)				
N	142	79	63	
Median(IQR)	50 (20–646)	50 (20–131)	88 (20–5990)	0.02^a^
≤ 50	85 (59.9%)	55 (69.6%)	30 (47.6%)	0.008^b^
> 50	57 (40.1%)	24 (30.4%)	33(52.4%)	
≥ 10 × 5	31 (37.8%)	14 (35%)	17 (40.5%)	
Cumulative length of undetectable VL (month)				
N	125	68	57	
Median (IQR)	12 (3–26)	19 (7–42)	5 (0.5–17)	< 0.001^a^
N	107	63 (100%)	44 (100%)	0.04^b^
Number of patient having undetectable VL less than 1 year	46	22 (34.9%)	24 (54.9%)	
Number of patient having undetectable VL more than 1 year	61	41 (65.1%)	20 (45.5%)	
Maximal VL (copies/ml)				
N	145	79 (100%)	66 (100%)	
Median (IQR)	64,600 (6170–1,87,000)	28,000 (2550–1,20,000)	1,00,000 (1675–2,71,250)	0.002^a^
< 10 × 5	81 (55.9%)	52 (65.8%)	29 (43.9%)	0.08^b^
≥ 10 × 5	64 (44.1%)	27 (34.2%)	37 (56.1%)	
Nadir CD4 at time of dysplasia (cells/mm^3^)				
N	145	79 (100%)	66 (100%)	
Median(IQR)	207 (102–317)	230 (171–345)	168 (78–290)	0.01^a^
≥ 200	75 (51.7%)	57 (62.7%)	28 (37.3%)	0.04^b^
< 200	70 (48.3%)	33 (40.5%)	38 (57.6%)	
≥ 100	110 (75.9%)	66 (83.5%)	44 (66.7%)	0.01^b^
< 100	35 (24.1%)	13 (16.5%)	22 (33.3%)	
≥ 50	124 (85.5%)	69 (87.3%)	55 (83.3%)	0.49^b^
< 50	21 (14.5%)	10 (12.7%)	11 (16.7%)	
CD4/CD8				
N	140	76	64	
Median (IQR)	0.5 (0.25–0.8)	0.6 (0.3–0.85)	0.32 (0.2–0.55)	< 0.001^a^
CD4 at the time of the dysplasia (cells/mm^3^)				
N	142	78 (100%)	64 (100%)	
Median (IQR)	430 (237–556)	491 (380–636)	320 (182–465)	< 0.001^a^
0–200	28 (19.7%)	9 (11%)	19 (29.7%)	< 0.001^b^
200–349	27 (19%)	9 (11.5%)	18 (28.1%)	
350–499	37 (26.1%)	23 (29.5%)	14 (21.9%)	
> 500	50 (35.2%)	37 (47.4%)	13 (20.3%)	
Management of the cervical dysplasia				
N	146	80 (100%)	66 (100%)	< 0.001^b^
Follow-up	15 (10.3%)	3 (3.8%)	12 (18.2%)	
Conisation	78 (53.4%)	59 (73.8%)	19 (28.8%)	
Ablative or local treatment	48 32.9%)	14 (17.5%)	34 (51.5%)	
Hysterectomy	5 (6.5%)	4 (5%)	1 (1.5%)	

*VL* viral load; *IVD* intravenous drug use; *cART* combination antiretroviral therapy; *HIV* human immunodeficiency virus

^a^Mann–Whitney test

^b^khi-carré test

Most HIV positive women had been infected through heterosexual contact and had been diagnosed with HIV at a median age of 28 years. Eighty-two percent had used cART, on average, for 34 months and 15% had already reached AIDS stage C (Table 4). Among women under cART, the most used cARTs were 2 NRTI (Nucleoside analog reverse-transcriptase inhibitors  +  1 PI (Protease Inhibitors; 54.8%), 2 NRTI  +  1 NNRTI (Non-nucleoside reverse-transcriptase inhibitors (23.3%) and 2 NRTI + 1 INTI (Integrase Inhibitos; 6.4%).

HIV-positive women, with successful cervical treatment had better HIV disease control: they had longer period of undetectable viral load (VL) (19 versus 5 months; p  <  0.001) than those with treatment failure. They also more frequently had VL  <  50 copies/ml at the time of the cervical treatment (69 versus 47%; p  =  0.008), lower peak VL between time of HIV diagnosis and cervical treatment (28,000 versus 1,00,000 copies/ml; p  =  0.002), higher nadir CD4 counts (230 versus 168 cells/mm^3^; p = 0.01) and a CD4/CD8 ratio that was significantly higher (0.6 versus 0.32; p  <  0.001).

Median CD4 count at time of dysplasia was 491 CD4  +  -lymphocytes cells/mm^3^ in women with no treatment failure versus 320 in women with failure (p  <  0.01). HIV-positive women who developed recurrence or persistence had been more often managed with local or ablative therapy (p  <  0.05). We analysed the odds ratio of having treatment failure among HIV patients. A multivariate analysis showed that HIV-positive women with a VL  >  50 copies at the time of dysplasia had 3, 5-fold (95% IC: 1.5–8.3) increased risk of treatment failure (p  =  0.004). Independently, being treated with local or ablative therapy rather than having had a conization was associated with a 7.4, four-fold (95% IC: 3.2–17.3) raised risk of having a treatment failure (p  <  0.001).

**Fig. 2:**
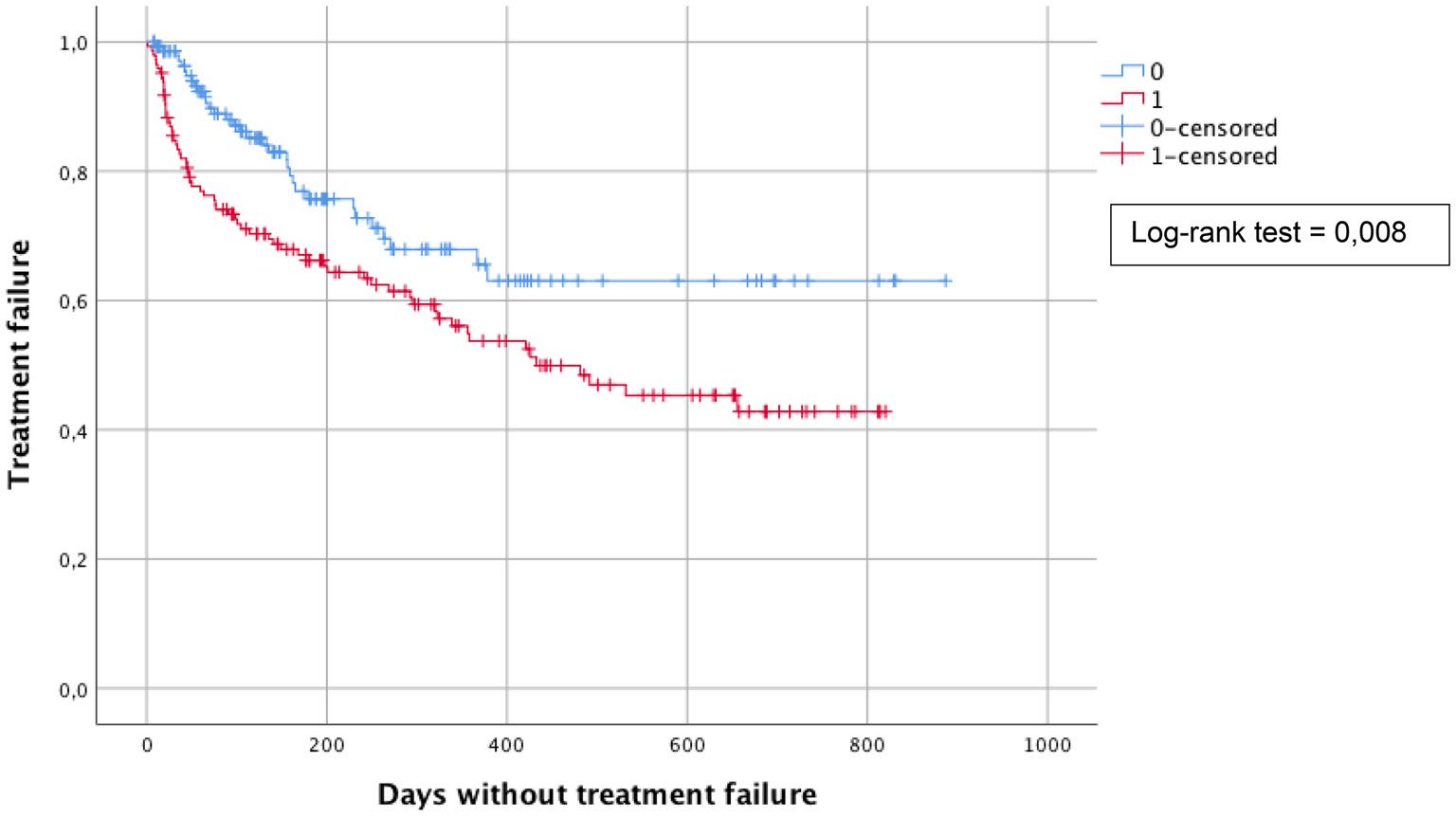
Kaplan–Meier survival curves representing treatment failure in HIV-positive (blue line) and negative (red line) women. Crosses represent normal follow-up smears (censored  =  no treatment failure) while decreases of the curve represent a treatment failure. HIV-positive women had more often treatment failure than HIV-negative ones. (log-rank test  =  0.008)

We analysed treatment failure among HIV-positive women versus HIV-negative ones using the Kaplan–Meier survival curve and observed that HIV-positive women had more recurrence or persistence than HIV-negative ones. (Log-rank test  =  0.008). Mean duration of time to treatment failure was 461 ± 31 days in HIV + women versus 620 ± 39  days in HIV negative ones (Fig. 2).

**Fig. 3:**
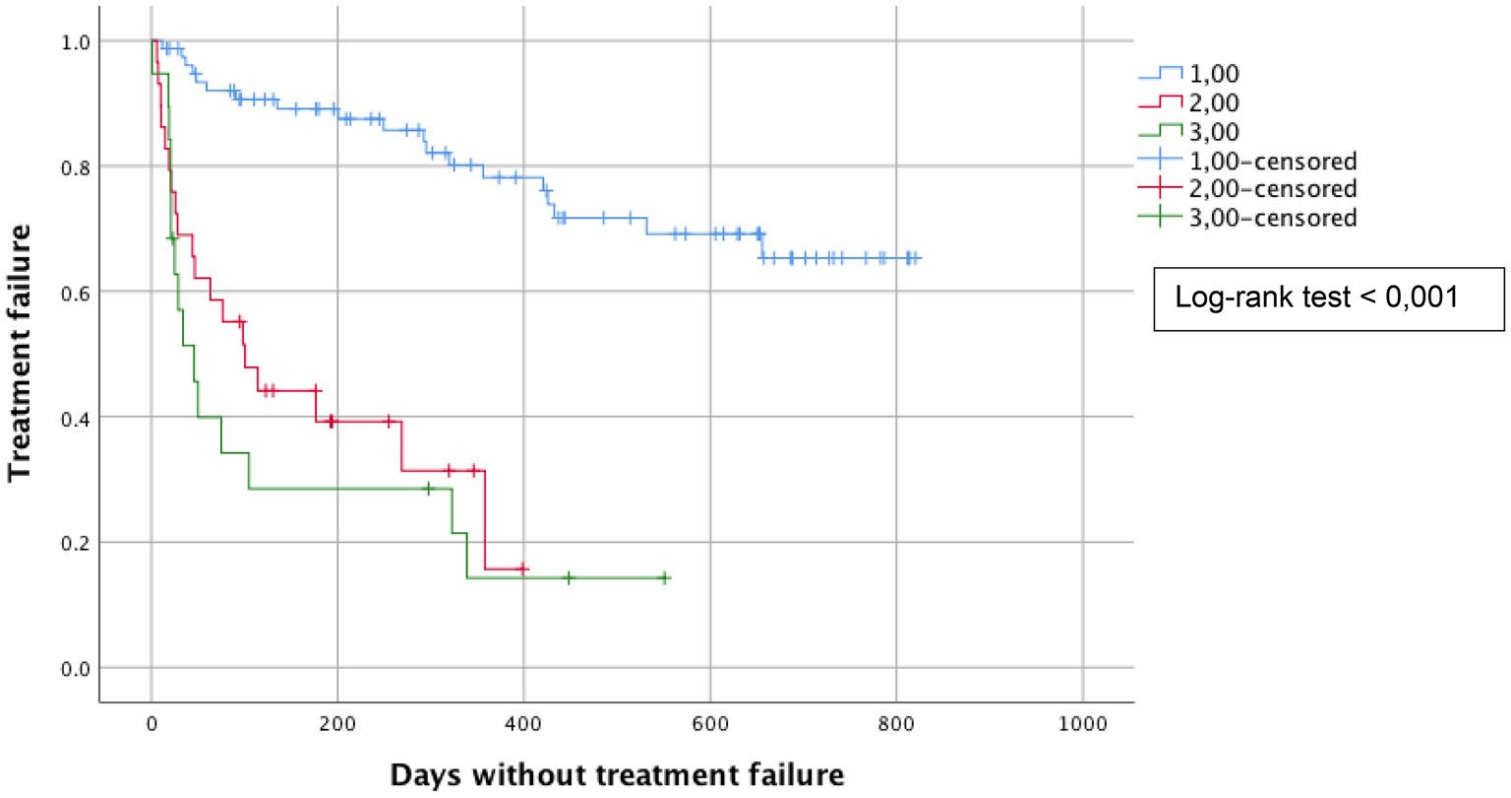
Kaplan Meyer survival curves representing treatment failure in relation to the type of treatment (ablative in red/local in green or excisional treatment in blue) in the total combined population of HIV-positive and negative women. Crosses represent normal follow-up smears (censored  =  no treatment failure) while decreases of the curve represent a treatment failure. The risk of treatment failure is higher when an ablative or local therapy is used compared to an excisional procedure (log-rank test  <  0.001)

We also analyzed treatment failure in relation to the type of treatment (ablative, local or excisional) and observed that the risk of treatment failure is higher when ablative or local therapy is used compared to excisional procedures (log-rank test < 0.001). Mean time to recurrence or persistence was 208 ± 22 days for ablative therapy, 211 ± 42 days for local therapy versus 695 ± 29 days for cone biopsy. (Fig. 3).

Being HIV positive was associated in the Cox regression model, with a significant increased risk of treatment failure (Hazard ratio 1.7; 95% confidence interval 1.1–2.6; p = 0.012). Similarly, ablative therapy was associated with an increased risk of treatment failure as compared to conization (Hazard ratio 1.2; 95% confidence interval 1.0–1.4; p = 0.012).

## Discussion

The aim of this study was to compare the management and outcome of high grade SIL in HIV-positive versus HIV-negative women.

Whereas the management of HSIL in the two groups was comparable, we observed that, after treatment of the cervical lesion, HIV-positive women had a 2.5 times higher risk of experiencing treatment failure. Our data are in concordance with a recent meta-analysis reporting that HIV-positive women had a two-fold higher risk of treatment failure compared to HIV negative women [8]. We also studied whether this treatment failure was due to either an increased persistence or an increased recurrence, rate. No differences were observed in terms of recurrence but 2.5 times increase in persistence risk was observed in HIV-positive women versus HIV-negative ones.

We observed, in both groups, a higher rate of treatment failure following local or ablative therapy than after LEEP. The data found in the literature are conflicting. In HIV-negative women, a meta-analysis comparing LEEP, cryotherapy and cold knife cone biopsy for HSIL, reported similar recurrence rates after 12 months, for LEEP and cryotherapy (5.3%) and a recurrence rate of 1.4% using cold knife cone biopsy [10]. The authors of this meta-analysis also included observational studies because of the paucity of randomized controlled trials (RCT) [10]. Another meta-analysis, including four RCTs in HIV-negative and -positive women, comparing cryotherapy and LEEP for the treatment of CIN 1, 2 or 3, found that treatment with LEEP was associated with a lower risk of persistence at 6 months (RR 0.87, 95% CI 0.76–0.99) and recurrence at 12 months (RR: 0.91, 95% CI 0.84–0.99) compared to cryotherapy treatment [11].

In our study, HIV-positive women, who had been treated by local or ablative therapy, had an increased risk of treatment failure. Similarly, two RCTs in HIV positive women, reported that cryotherapy was associated with a higher rate of treatment failure than LEEP. Chirenje et al. [12] observed a treatment failure after cone biopsy of 4% (n  =  50) versus 14% after cryotherapy (n  =  42) at the12 month follow-up. Green et al. [13] compared 200 HIV-positive women who had HSIL and were treated with cryotherapy, to 200 treated using cone biopsy, and observed a treatment failure rate of 30% in the cryotherapy group versus 19% in the biopsy group after 24 months. On the other hand, a randomized study found no difference after 12 months, between 86 women who had had a cone biopsy for HSIL and 80 women treated by cryotherapy [14]. In the meta-analysis of Debeaudrap et al. [8] a sub-group analysis limited to HIV-positive women treated for HSIL showed a higher risk of treatment failure after cryotherapy (21.6%) compared to cone biopsy (12.6%).

Although the size of the removed cones was similar between HIV-positive and -negative women in our study, the margins were more often positive in HIV-positive women (37 versus 16%). These observations were also found in other studies and support the idea of the presence of more extensive lesions in HIV-positive women compared to HIV-negative ones and could explain treatment failure [8].

Comparing HIV women with treatment failure and those without, we observed that shorter duration of cumulative undetectable HIV VL, or VL  >  50 copies/ml at the time of HSIL treatment, higher maximum VL between time of HIV diagnosis and cervical excision, a lower CD4/CD8 ratio, lower nadir CD4 count and lower CD4 count at time of dysplasia were associated with higher rates of treatment failure. Atemnkeng et al. [15] evaluated the association between cART, viral load, CD4 count and detection of CIN 2  +  at follow-up. They observed that cART intake was associated with a reduced risk of CIN 2  +  detection at follow-up, after excisional procedures, but not after ablative treatment, as well as in cART users with undetectable VL. They did not report an association between CD4 count at the time of dysplasia discovery and treatment failure. Two studies, within the meta-analysis of Atemnkeng, reported that a nadir CD4 count of more than 350 cells/mm^3^ was associated with a 65% decreased risk of CIN 2  +  detection at follow-up.

At this time, the recommendations for HSIL management between HIV positive and negative women do not differ. The World Health Organization and ASCCP recommend excision over ablation in settings where both are available, but ablation is acceptable for young women in satisfactory clinical conditions (when pregnancy is desired, the colposcopy image is satisfactory and follow-up compliance is probable) [10, 16].

In our clinical practice, managing young, HIV positive women with a recent diagnosis of HSIL and control of their HIV infection presents a challenge, when weighing the potential, future obstetric risk against the risk of cervical lesion progression.

Indeed, despite the scaling up access of cART and easier regimens (one pill regimen with less side effects), invasive cervical cancer is still more prevalent in HIV-positive women [17]. A recent study reported data regarding cervical cancer risk, in women living with HIV across four continents. Being older than 50 and having a low CD4 count at the time of cART initiation, were risk factors for invasive cervical cancer development [17]. Our data are in agreement, since we found that cervical treatment failure was 3.5 times higher in women who had a viral load exceeding 50 copies/ml at the time of the HSIL diagnosis.

We therefore suggest that cervical cancer management guidelines must be adapted for HIV-positive women. Excisional treatment should be preferred over ablative therapy, particularly in women with uncontrolled viral load at the time of the HSIL diagnosis. Ablative therapy could be an option for young women who have an undetectable viral load and CD4  +  lymphocytes higher than 500 cells/mm^3^ and have a desire of pregnancy.

In our study, few patients were vaccinated against HPV, even though it is known that HPV vaccination at the time of the cone biopsy, reduces the risk of recurrence of HSIL in HIV-negative women [18, 19]. Although this has not yet been established in HIV-positive women, similar results, for them, might be found. In view of the high recurrence rate, studies should be performed to find an answer to this question. Pending their results, vaccination should be proposed for both HIV-negative and positive women.

Our study has several limitations, mostly related to the retrospective design and the real clinical setting of a cohort, so we were not able to match the two groups for all of the possible confounding factors. Most of the women in the HIV group were Black African patients and most of our HIV negative patients were of European origin. This is an important confounder that we could not correct for a number of variables, results were occasionally unavailable. Not all women had the same follow-up schedule. In some cases, distinguishing persistence from a re-infection may have been erroneous. Moreover, the guidelines on ART and the identification of high risk HPV (HR HPV) has evolved over time. Finally, vaccination has only been available since 2008 in Belgium. However, the large cohort with a long follow up, the detailed HIV infection surrogates markers and the real-life follow up setting contribute to the strengths of this study.

## Conclusion

HIV-positive women have a higher rate of treatment failure for HSIL than do HIV-negative women. Ablative therapy is associated with a higher risk of treatment failure, especially in women with poor control of their HIV infection and should therefore be avoided for these patients. The management and the follow- up of HSIL’s guidelines in this high-risk population should be adapted consequently: for HIV-positive women with uncontrolled viral load, excisional treatment should be the preferred therapy, whereas for women with undetectable viral load, CD4  +  lymphocytes higher than 500 cells/mm^3^ and with a desire of pregnancy, ablative therapy may be considered.

## Data Availability

Not applicable.
